# The analysis of *DMD* gene deletions by multiplex PCR in Indonesian DMD/BMD patients: the era of personalized medicine

**DOI:** 10.1186/s13104-019-4730-1

**Published:** 2019-10-28

**Authors:** Kristy Iskandar, Ery Kus Dwianingsih, Linda Pratiwi, Alvin Santoso Kalim, Hasna Mardhiah, Alifiani H. Putranti, Dian K. Nurputra, Agung Triono, Elisabeth S. Herini, Rusdy G. Malueka, Gunadi, Poh San Lai, Sunartini

**Affiliations:** 1 Department of Child Health/Genetics Working Group, Faculty of Medicine, Public Health and Nursing, Universitas Gadjah Mada/UGM Academic Hospital, Jl. Kabupaten (Lingkar Utara), Kronggahan, Trihanggo, Gamping, Sleman, Yogyakarta, 55291 Indonesia; 2 Department of Anatomical Pathology/Genetics Working Group, Faculty of Medicine, Public Health and Nursing, Universitas Gadjah Mada/Dr. Sardjito Hospital, Yogyakarta, 55281 Indonesia; 3 Genetics Working Group, Faculty of Medicine, Public Health and Nursing, Universitas Gadjah Mada, Yogyakarta, 55281 Indonesia; 4 Department of Child Health, Faculty of Medicine, Universitas Diponegoro/Dr. Kariadi Teaching Hospital, Semarang, 50244 Indonesia; 5 Department of Child Health, Faculty of Medicine, Public Health and Nursing, Universitas Gadjah Mada/Dr. Sardjito Hospital, Yogyakarta, 55281 Indonesia; 6 Department of Neurology/Genetics Working Group, Faculty of Medicine, Universitas Gadjah Mada/Dr. Sardjito Hospital, Yogyakarta, 55281 Indonesia; 7 Pediatric Surgery Division, Department of Surgery/Genetics Working Group, Faculty of Medicine, Universitas Gadjah Mada/Dr. Sardjito Hospital, Yogyakarta, 55281 Indonesia; 8 Department of Pediatrics, Yong Loo Lin School of Medicine, National University of Singapore, Singapore, 119074 Singapore

**Keywords:** Duchenne/Becker muscular dystrophy, *DMD* gene deletion, Exon skipping therapy, Multiplex PCR

## Abstract

**Objective:**

Duchenne/Becker muscular dystrophy (DMD/BMD) is the most common genetic neuromuscular disease in children, resulting from a defect in the *DMD* gene located on Xp21.2. The new emerging treatment using exon skipping strategy is tailored to specific mutations, thus molecular diagnostics are particularly important. This study aimed to detect the *DMD* gene deletion in Indonesian DMD/BMD patients and analyze the potential amenability by exon skipping therapy.

**Results:**

Thirty-four male patients were enrolled in this study, 23 of them (67.6%) underwent muscle biopsy and showed the absence or partially expressed dystrophin protein in immunohistochemistry staining. All patients had very high serum CK levels (10.529 ± 9.97 IU/L). Multiplex PCR revealed the *DMD* gene deletions in 15 (44.1%) cases. Seventy-eight percent of deletions were clustered in the hot-spot region of exon 43 to 52. Furthermore, seven (20.5%) patients were potentially amenable to exon skipping treatment. Therefore, multiplex PCR is one feasible method to detect *DMD* gene deletion in Indonesian DMD/BMD patients that can further determine the potential amenability of exon skipping therapy. In addition, this study is the first report of *DMD* gene deletion analysis in Indonesia.

## Introduction

Duchenne and Becker muscular dystrophies (DMD; MIM 310200/BMD; MIM 300376) are X-linked recessive neuromuscular disorders. DMD is the most common and most severe form, with an incidence of one in 3500–5000 live male births [1]. DMD/BMD is caused by mutations in the *DMD* gene on the Xp21.2 region (MIM 300377). The *DMD* gene is the largest human gene, consisting of 79 exons that encode a 14 kb mRNA and produce the 527 kDa dystrophin protein, a cytoskeletal protein than enables the strength, stability and functionality of myofibres [2].

Progressive muscular damage occurs in patients with DMD, resulting in muscular weakness, associated motor delays, loss of ambulation, respiratory impairment, and cardiomyopathy. DMD patients are usually diagnosed by the age of 5, and wheel chair dependent before the age of 13. Without intervention, death usually occurs within two decades of life, as a result of cardiac and respiratory compromise [2]. BMD is a milder form of DMD, and patients are usually capable of walking independently until the age of 16 years or later and have a normal life expectancy [1].

Most identified mutations in DMD patients are large deletions (65%), followed by point mutations (26%), duplications (7%), and others 2% (including intronic, or 5′ and 3′ UTR alterations) [3–5]. Mutations are either inherited from asymptomatic female carriers (~ 70%) or de novo (~ 30%) [6]. Frame shift (out of frame) mutations will result in a DMD phenotype, while in frame mutations will result in a BMD phenotypes.

The underlying pathology in DMD patients is the absence of dystrophin caused by mutation. Therefore, emerging therapies are aimed to restore muscle dystrophin. Exon skipping is a potential method to restore some of dystrophin protein and thus a promising therapy for DMD [7]. Other dystrophin restoration therapies are in development and some are near or in regulatory review [8].

Genetic testing for diagnosis is important to allow patients appropriate planning of care and treatment. International working groups for DMD standard of care [2, 9] have recommended the genetic testing bypassing muscle biopsy, which is a common diagnostic procedure in tertiary hospitals in Indonesia. Herein, we provide the first report on description of *DMD* gene deletions in Indonesia by using multiplex PCR. The study also assesses the eligibility for potential exon skipping therapy for DMD patients.

## Main text

### Methods

#### Patients

Thirty-four male patients from Dr. Sardjito Teaching Hospital and Universitas Gadjah Mada (UGM) Academic Hospital, Yogyakarta, Indonesia were enrolled in this study. All patients fulfilled the DMD/BMD diagnostic criteria based on clinical presentation and biochemical analysis. In most patients (67.6%), diagnosis was confirmed by immunohistochemistry with dystrophin protein staining. Written informed consent for genetic study was obtained from the parents. The study protocol was approved by the ethical committee of Faculty of Medicine, Public Health and Nursing UGM (KE/FK/1164/EC/2017).

#### Genomic DNA extraction

Genomic DNA was isolated from 3 mL of EDTA-peripheral whole blood samples using Qiagen^®^ QIAamp DNA Mini Kit according to manufacturer protocol.

#### Molecular analysis

Multiplex PCR was performed as described by Abbs et al. [10], Chamberlain [11] and Beggs et al. [12]. The tested exons were 3, 4, 6, 8, 12, 13, 17, 19, 43, 44, 45, 46, 47, 48, 49, 50, 51, 52, 53, 60 and muscle specific promoters which cover the hot spots region in the *DMD* gene. We made 6 sets of reaction mixture. The reaction mix (25 μL) contained 100 ng genomic DNA, 1× PCR buffer, 20 μmol/L of each primers and Go Taq^®^ Master mix (Promega, Madison, WI, USA). Set I primers flanked exons 4, 12, 17, 44, and 60; set II flanked exons 8, 13, 6, and 52; set III flanked exons 50, 51, and dpm 427; set IV flanked exons 3, 45, 46, 47, set V flanked exon 48 and set VI flanked exon 49 and 53. Cycling conditions were 35 cycles of denaturation at 94 °C for 1 min, annealing at 50 °C for 1 min, and extension at 72 °C for 5 min (Set I). The cycling conditions were the same for other sets except the annealing temperature were 56 °C and 58 °C for set II and III respectively, 52 °C for both set IV and V, and 60 °C for the set VI. All reactions were carried out using ProFlex PCR System (Applied Biosystems). 10 μL PCR products and a DNA size ladder were electrophoresed in 2% agarose gel stained with 0.5 μg/mL Fluorosafe™, visualized and photographed on an ultraviolet transilluminator.

#### Reading frame analysis

The reading frame was analysed using the online DMD exonic deletion reading frame checker version 1.9, available at: http://www.dmd.nl from The Leiden Muscular dystrophy.

### Results

#### Clinical results

In this study, we analyzed 34 DNA samples from DMD/BMD patients. The most common presenting symptoms were frequent falls, muscle weakness and abnormal gait. Age of initial symptoms was noticed at 4.8 ± 2.1 years. The mean age at diagnosis was 6.8 ± 2.8 years. First walking was reported on average at 16 ± 4 months of age (11–30 months). Thirteen patients (38.2%) have family history of DMD/BMD. All patients had very high serum CK levels (10.529 ± 9.97 IU/L). Twenty-three (67.6%) patients underwent muscle biopsies. Twenty-one patients showed a complete absence and 2 patients showed partial absence of dystrophin via immunohistochemical analysis (Table 1).

**Table 1:** Baseline characteristics of subjects

ID no	CK level (IU/L)	Onset age (years)	Age at diagnosis (years)	Age at first walk (months)	Family history	Deletion status
1	4906	6	7	18	No	Negative
2	3022	1	1	18	Yes	Negative
3	4135	1	1	18	Yes	Negative
4	1749	1	1	12	Yes	Negative
5	10,499	5	8	13	No	Negative
6	17,388	3	3	13	Yes	Positive
7	2677	8	10	13	Yes	Positive
8	2020	4	4	11	No	Negative
9	3932	5	14	13	No	Positive
10	11,152	5	7	13	No	Positive
11	16,550	4	6	13	No	Positive
12	10,846	5	8	18	No	Negative
13	3125	8	9	13	No	Positive
14	8871	4	9	12	No	Positive
15	6239	5	6	13	No	Positive
16	3255	6	9	18	No	Positive
17	6924	5	6	13	Yes	Negative
18	12,948	7	10	30	No	Negative
19	28,060	6	7	18	No	Negative
20	N/A	6	8	16	Yes	Negative
21	N/A	6	7	12	Yes	Negative
22	11,433	5	7	24	Yes	Positive
23	N/A	1	4	18	No	Positive
24	40,429	6	6	14	Yes	Negative
25	8961	6	8	12	No	Negative
26	4137	9	9	18	No	Negative
27	8430	6	9	18	No	Negative
28	8751	4	4	24	No	Positive
29	38,966	3	7	24	Yes	Positive
30	20,480	3	5	15	Yes	Positive
31	7482	3	8	14	No	Negative
32	6746	8	9	13	No	Positive
33	5448	6	9	13	Yes	Negative
34	6857	4	7	21	No	Negative

#### Molecular results

**Fig. 1:**
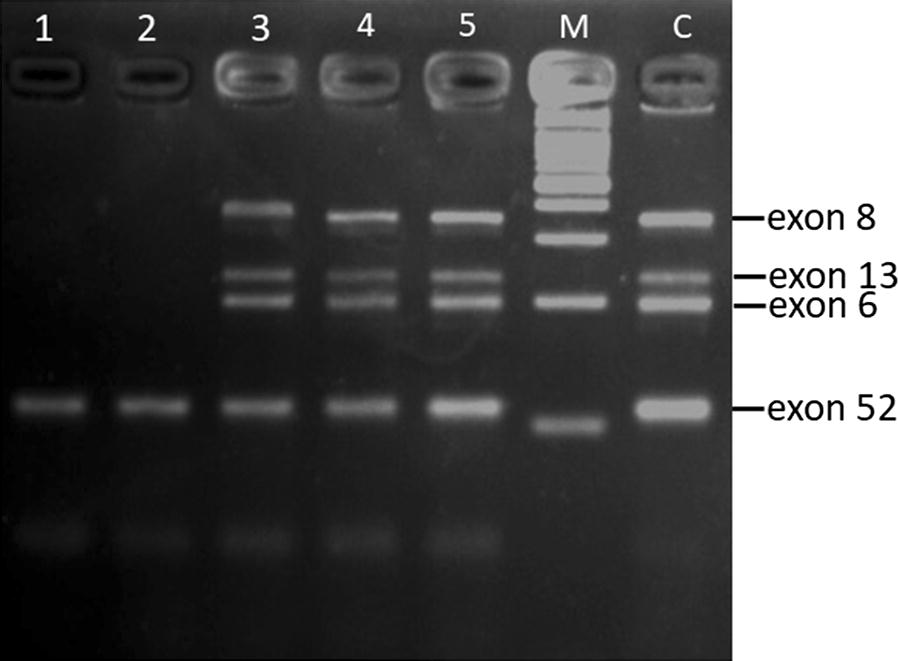
Multiplex PCR analysis: *DMD* gene deletion encompassing exons (*M* marker, *C* control normal patient, (1, 2, 3, 4, 5) patients with DMD). It is showed that patients no 1 and 2 had deletions in exons 8, 13, 6 but not 52

Deletion was detected in 15 out of 34 DMD/BMD patients (44.1%) by multiplex PCR (Fig. 1).

All deletions were found clustered in two deletions-prone regions. Among the 15 deletions, 12 (78%) were found in exon 43 to 52, 1 deletion in the proximal region, and 2 mutations covering both hotspots region (Additional file 1: Figure S1).

The DMD reading-frame checker 1.9 from The Leiden Muscular dystrophy website (https://www.dmd.nl) predicted out of frame mutations in 14 (92.8%) patients and in frame mutation in 1 (0.07%) patient. Most of the out of frames mutations predicted were in accordance with dystrophin staining, except in 1 patient. He has partial absence of dystrophin, but the reading frame analysis showed out of frame mutation.

Based on Duchenne population potentially amenable to exon skipping prepared by CureDuchenne [13], our results showed that 7 out of 15 (40%) patients with detected deletions are potentially amenable to exons skipping therapy. Four patients for exon 53 skipping, and 3 patients will be potentially eligible for exon 51 skipping (eteplirsen; exondys51^®^). Although the deletion in patient no 9 (NPP) had amenability to exon 45 skipping therapy, but he had clinically BMD (Table 2). The patient also showed some production of dystrophin in the muscle, therefore exon skipping therapy would not benefit in this case. Out of 34 subjects tested, exon 53 skipping and exon 51 skipping would be beneficial in 11.7% and 8.8% of DMD patients.

**Table 2:** Data of muscle biopsy with dystrophin staining, deletion detected by multiplex PCR, reading frame, and eligibility to exon skipping therapy

ID no	Dystrophin staining	Exons deletions	Reading frame	Amenability to exon skipping
1	Absence	12, 17, 44	Out of frame	N/A
2	N/A	3–17, 44	Out of frame	N/A
3	N/A	3–17, 44	Out of frame	N/A
4	N/A	8–17	Out of frame	N/A
5	N/A	45–52	Out of frame	Exon 53 skip-amenable
6	Absence	45–52	Out of frame	Exon 53 skip-amenable
7	N/A (HE only)	46–50	Out of frame	N/A
8	N/A (HE only)	46–50	Out of frame	N/A
9	Partly	46–51	Out of frame	Exon 45 skip-amenable
10	Absence	48–50	Out of frame	Exon 51 skip-amenable
11	Partly	50–51	In frame	N/A
12	N/A (HE only)	50	Out of frame	Exon 51 skip-amenable
13	N/A	50	Out of frame	Exon 53 skip-amenable
14	Absence	50–52	Out of frame	Exon 53 skip-amenable
15	Absence	52	Out of frame	Exon 51 skip-amenable

### Discussion

DMD is a devastating progressive neuromuscular disease, for which, in Indonesia there is currently no effective treatment except palliative therapy and corticosteroids which have been proved to prolong the progressivity of the disease [14]. Recently, promising genetic therapies have been developed, to target and restore dystrophin in myocytes from patients, offering hope for the patients [8, 15, 16]. There are several approaches, including viral delivery of the missing *DMD* gene, read-through of translation stop codons, exon skipping to restore the reading frame and increased expression of the compensatory *utrophin* gene [15]. Exon skipping therapies with antisense oligonucleotides (AOs) is a promising therapy for DMD, and is currently the focus of clinical trials [8, 16]. It uses antisense oligonucleotides to splice out selected exons from the pre-mRNA at or next to the mutation site, to generate a translatable transcript from the mutant *DMD* gene, which are partially functionally similar to milder dystrophinopathy Becker muscular dystrophy [17]. It means that it is a treatment but not a cure. Clinical trials targeting exons 44, 45, 51 and 53 are being globally investigated and near regulatory approval [8]. Overall the exon skipping therapy may eventually apply to 60–80% DMD patients [13]. Eteplirsen are the first of a series mutation specific therapies to gain regulatory approval by US Food and Drug Administration in 2016. It targets approximately 13% of DMD patients that have *DMD* gene mutation amenable to exon 51 skipping, however it can be costly and may not be available in certain countries including Indonesia [18].

Detection of *DMD* deletion mutation is important in the diagnosis of DMD. In the present study, multiplex PCR can detect 44.1% of *DMD* gene deletions. Our data also revealed that 7 out of 34 (20.6%) DMD patients in this study would benefit from exon skipping therapies, with 3 potentially amenable with FDA approved drug, eteplirsen.

Our data were similar to the reported deletion rates using multiplex PCR in Asian population; 40%, 51.2%, 32.4% and 49% in Singapore, Japanese and Vietnamese and Thailand patients, respectively [19, 20]. In accordance with our findings, others have shown that ~ 20–30% of detected deletion clusters were in the proximal hotspot and ~ 70–80% in the distal hotspot [10]. The reading-frame hypothesis holds for > 90% of cases and can guide the early-stage clinical evaluation of DMD and BMD patients. In our study, we found 93.3% cases were consistent with the reading frame rule. One patient did not meet the rule, which could be caused by alternative translation initiation site that produced truncated dystrophin detectable by immunohistochemistry staining [21], despite being predicted to be an out of frame mutation [6]. Further analysis to know the exact sequences is needed.

The accurate multiplex PCR techniques are useful in the initial step of molecular diagnosis of DMD and BMD. The deletion rate is the same worldwide; i.e. about 65%, and undetected deletion may be due to multiplex PCR primers that do not capture all the exons continuously [22]. MLPA (multiple ligation-dependent probe) screens all 79 exons and allows to define the deletion breakpoints of the exons. Additionally, it allows detection of duplication of exons and carrier state which multiplex PCR does not. However, the availability of capillary electrophoresis is limited in Indonesia, and also it is much more expensive than multiplex PCR.

### Conclusion

The multiplex PCR method is an effective diagnostic tool for DMD/BMD screening, especially in settings with limited resources. It is a simple, rapid, non-invasive and cost-effective approach. Specific mutation data are mandatory for future approved genetic therapies. The Indonesian DMD/BMD registry with mutation analysis data is needed not only to know the eligibility for genetic therapies, but also to provide better genetic counseling, carrier testing, and prenatal screening.

## Limitations

The multiplex PCR cannot detect all deletions in the *DMD* gene nor any duplications or point mutations, therefore a negative result cannot rule out the possibility of DMD diagnosis. We also realized of the limitations in our relatively small sample size and limited geographical distribution amongst the immensely diverse Indonesian population therefore further multicenter research with larger sample size is needed to confirm our findings.

## Supplementary information

**Additional file 1: Figure S1.** Graphical representation of distribution of deletions in the *DMD* gene in DMD/BMD patients. Exons numbers for the *DMD* gene are indicated at the top. The number of cases having deletions is indicated in parenthesis. Deletions is showing in two major hot spots.

## Data Availability

All data generated or analyzed during this study are included in the submission. The raw data are available from the corresponding author on reasonable request.

## Abbreviations

AOs: antisense oligonucleotides
BMD: Becker muscular dystrophy
CK: creatinine kinase
DMD: Duchenne muscular dystrophy
DNA: deoxyribonucleic acid
EDTA: ethylenediamine tetraacetic acid
mRNA: messenger ribonucleic acid
PCR: polymerase chain reaction
RNA: ribonucleic acid
UTR: untranslated region
