# Supplementary figures and images for: The analysis of DMD gene deletions by multiplex PCR in Indonesian DMD/BMD patients: the era of personalized medicine
Source: BMC Res Notes. 2019 Oct 28;12:704. doi: 10.1186/s13104-019-4730-1 (PMC6819651; doi:10.1186/s13104-019-4730-1)

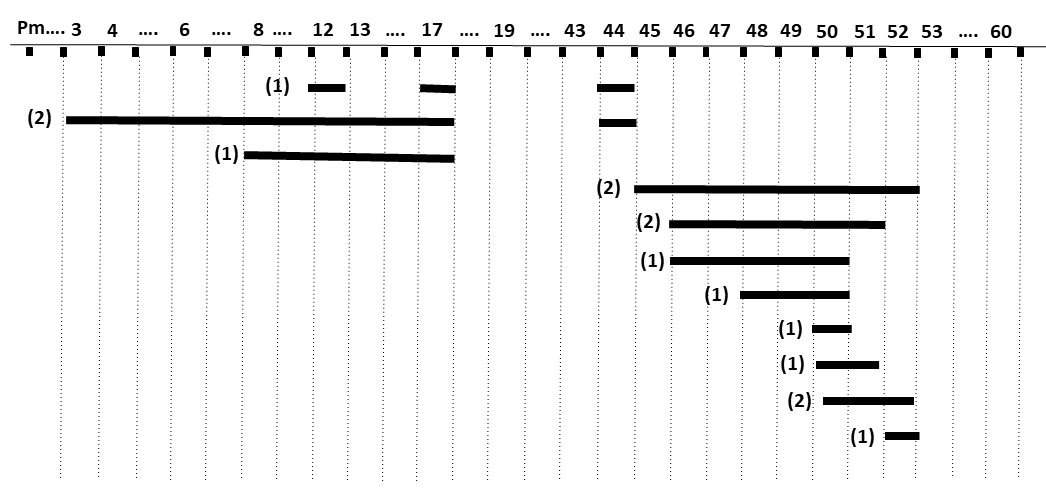

Supplement: Supplementary file 1 — Additional file 1: Figure S1. Graphical representation of distribution of deletions in the DMD gene in DMD/BMD patients. Exons numbers for the DMD gene are indicated at the top. The number of cases having deletions is indicated in parenthesis. Deletions is showing in two major hot spots. [file 13104_2019_4730_MOESM1_ESM.tif]
